# Heroin Use Is Associated with Suppressed Pro-Inflammatory Cytokine Response after LPS Exposure in HIV-Infected Individuals

**DOI:** 10.1371/journal.pone.0122822

**Published:** 2015-04-01

**Authors:** Hinta Meijerink, Agnes Indrati, Fitri Utami, Suharyani Soedarmo, Bachti Alisjahbana, Mihai G. Netea, Reinout van Crevel, Rudi Wisaksana, Andre Jam van der Ven

**Affiliations:** 1 Department of Internal Medicine, Radboud University Medical Center, Nijmegen, the Netherlands; 2 Health Research Unit, Faculty of Medicine, Universitas Padjadjaran/Hasan Sadikin Hospital, Bandung, Indonesia; Temple University School of Medicine, UNITED STATES

## Abstract

**Background:**

Opioid use is associated with increased incidence of infectious diseases. Although experimental studies have shown that opioids affect various functions of immune cells, only limited data are available from human studies. Drug use is an important risk factor for HIV transmission; however no data are available whether heroin and/or methadone modulate immune response. Therefore, we examined the effect of heroin and methadone use among HIV-infected individuals on the production of cytokines after *ex vivo* stimulation with various pathogens.

**Methods:**

Treatment naïve HIV-infected individuals from Indonesia were recruited. Several cohorts of individuals were recruited: 1) using heroin 2) receiving methadone opioid substitution 3) using heroin over 1 year ago and 4) controls (never used opioids). Whole blood was stimulated with *Mycobacterium tuberculosis*, *Candida albicans* and LPS for 24 to 48 hours. Cytokine production (IL-1 β, IL-6, IL-10, IFN-α, IFN-γ and TNF-α) was determined using multiplex beads assay.

**Results:**

Among 82 individuals, the cytokine levels in unstimulated samples did not differ between groups. Overall, heroin users had significantly lower cytokine response after exposure to LPS (p<0.05). After stimulation with either *M*. *tuberculosis* or *C*. *albicans* the cytokine production of all groups were comparable.

**Conclusion:**

The cytokine production after exposure to LPS is significantly down-regulated in HIV-infected heroin users. Interesting, methadone use did not suppress cytokine response, which could have implications guidelines of opioid substitution.

## Introduction

Opioid use is associated with increased incidence of infectious diseases. Variables, such as sharing needles, nutrition status and homelessness, can influence the frequency of infections, but studies also suggest that immune modulating effects of opioids are a major factor in the increased susceptibility to various infectious agents [1–8]. More specifically, opioids have shown to modulate several aspects of the immune system.

In both human and animal studies, opioid use has been associated with increased susceptibility to microbial infections, including bacterial infections such as staphylococci, streptococci and more specifically *Escherichia coli*, *Salmonella typhimurium* and *Mycobacterium tuberculosis*, the fungi *Candida albicans*, the parasite *Toxoplasma gondii*, and viruses such as the herpes virus and Friend leukemia virus [7]. In addition, there is a high risk of blood-born infectious diseases when unsterile needles are used to inject drugs, such as endocarditis, cellulitis, sepsis, hepatitis C and human immunodeficiency virus (HIV) [6]. Injecting drug use (IDU) is responsible for HIV infections in 10% of all cases worldwide, and 30% of cases outside Africa [1]. IDU is not only a risk factor for HIV transmission, but it may also change the natural course of HIV infection, for instance because of co-infections, nutritional deficiencies and/or the immune modulating properties of opioids.

Experimental studies have shown that binding of opioids to the opioid μ-receptors of immune cells affects the function of these cells in various ways. *In vitro* and animal experiments have shown that opioids modulate phagocytosis, chemotaxis, cytokine response and activity of immune cells; which could have detrimental results for the cellular immunity [6,7]. On the other hand, other studies suggest that the effects of opioids on the immune system are not necessarily deleterious; cells exposed to endogenous opioids *in vitro* increased natural killer cell activity [9].

The regulation of immune and inflammatory responses is dependent on the functions of cytokines, which is especially important in individuals with an already compromised immune function, such as HIV-infected individuals. The effects of opioids on cytokine production have been investigated in both *in vitro* and animal experiments [10–19], but only little information is available on *in vivo* exposure in humans. In addition, the data from previous studies have shown contradicting results. For example the production of IFN-γ can be enhanced [10] or suppressed [11] by various opioids in *in vitro* studies, whereas in animal experiments the IFN-γ production is often diminished after exposure to opioids [17,18]. The production TNF-α is mostly suppressed by opioids [13,15,16], but some studies show no effect [14] or even an increase in TNF-α [12]. Opioids reduce the IL-8 production [13,19], whereas IL-1β production is often enhanced [12,19]. The differences in results might be explained by experimental set-up, type of cells or animals used, and type of opioid.

Most studies use morphine to test the effects of opioids on immune system. Only limited data are available from human studies; in most of these studies opioids are used for pain relief, either post-surgery or during cancer treatment [20–24]. The main findings of these studies were that morphine suppressed natural killer cell activity, reduced mitogen responses and decreased lymphoproliferation. One study examined the effects of morphine in healthy volunteers [25]: continuous exposure to morphine over a period of 36 hours resulted in suppression of natural killer cell cytotoxicity as well as IFN-γ stimulated and antibody-dependent cytotoxity. All these study suggests that morphine administration can cause measurable suppression of the immune system.

Heroin and methadone are however more commonly associated with HIV infection. As the different opioids are known to have different affinity for the opioid receptors [26–28], one may hypothesize that they exert different immune modulating effects. Only two human studies examined the immunological effects of heroin or methadone. One study compared the effects of methadone and buprenorphine (as opioid substitution) in drug users and found no difference, but cytokine levels were significantly depressed compared to controls [29]. Azarang *et al* compared the *ex vivo* cytokine production after stimulation of whole blood between heroin users and healthy controls and found that heroin addicts produce less IFN-γ and more interleukin (IL)10 after stimulation with lipopolysaccharides (LPS) [30]. Even though drug use is not uncommon among HIV-infected individuals, no data are available regarding the effects of heroin or methadone on the immune response in HIV. In the present study, we examined therefore the effect of heroin and methadone use among HIV-infected individuals on the production of cytokines after *ex vivo* stimulation with various pathogens relevant in the HIV patients.

## Methods

### Study setting and population

For this study, we included HIV-infected adults (>16 years) from several settings in West-Java, Indonesia; an HIV clinic, a methadone clinic and the community. The collaboration with the clinics and the community was part of a five-year programme (2006–2011) called ‘IMPACT’, aimed to improve prevention, control and treatment of HIV among injecting drug users in West-Java, Indonesia [31]. In these clinics, people with and without a history of IDU, who are at risk for HIV infection or who present with signs and symptoms suggesting HIV/AIDS are counselled and tested for HIV. All testing is voluntary and informed consent is obtained from all study participants. HIV-infected patients are characterised and followed prospectively in a cohort study. To contact individuals in the community we collaborated closely with Rumah Cemara; a local NGO focussing on increasing the quality of life for people with HIV/AIDS and people who use drugs. All subjects selected for this study were antiretroviral treatment naïve HIV-infected and had no signs of active infections, such as acute hepatitis, active tuberculosis or oral thrush. Participants were not tested for (chronic) hepatitis C infection or latent tuberculosis. Individuals had to fit the inclusion criteria of one of the four groups: 1) Active drug users: used heroin in the last 30 days; 2) Methadone clients: received methadone maintenance treatment (MMT) in the last 30 days; 3) Former users: used heroin or methadone over one year ago; 4) Controls: never used heroin or methadone. All participants were informed on the study and signed an informed consent. Data on demographic factors, history of drug use, co-morbidity, self-reported tuberculosis treatment and history of ART were collected through interview with standardized questionnaires. In addition, blood was collected for various purposes, including CD4 cell count, general haematology, flow cytometry and cell stimulation assays. At the time of this study, antiretroviral therapy (ART) was indicated in Indonesia for patients presenting with WHO clinical stage IV or a CD4 count less than 200 cells/μl in accordance with WHO guidelines from 2006. Since 2004, ART can be accessed free of charge in Indonesia.

### Ethics Statement

After being informed about the study, all participants provided written consent to participate in this study. No children or minors were included in this study. This study was approved by the Health Research Ethics Committee at the Faculty of Medicine of Padjadjaran University/Dr. Hasan Sadikin General Hospital in Bandung, Indonesia (record number: 190/ UN6.C2.1.2/ KEPK/ PN/ 2011).

### Stimulation assay

Cytokine production was examined after whole blood stimulation with various pathogens, namely killed *Candida albicans* and *Mycobacterium tuberculosis* as infection with these are often associated with HIV and LPS isolated from *Escherichia coli* as a TLR4 agonist. Briefly, venous blood was drawn into 5-mL endotoxin-free lithium-heparin tubes (Vacutainer, BD Biosciences) and samples were processed within 4 hours. Blood was diluted 1:5 with culture medium only (RPMI Dutch modified) or in combination with either conidia of heath killed *C*.*albicans* (10^6^ cells/ml), *M*. *tuberculosis* (10 μg/ml), LPS isolated from *E*. *coli* (10 ng/ml). After 24 or 48 hour incubation, blood cultures were centrifuged at 1700rpm for 10 min and supernatants were stored at −20°C until assayed. During this period opioid levels were not measured, nor was the opioid level maintained during incubation. The expression of cytokines IL-1 β, IL-6, IL-10, IFN-α, IFN-γ and TNF-α were measured using a multiplex beads assay (Merck Millipore, Billerica, MA, USA) according to the manufacturer's instructions. The detection limit for the cytokine production was 32pg/μl for IL-1β and IL-6 and 3.2pg/μ for and IL-10, IFN-α, IFN-γ and TNF-α. Based on previous testing we measured the production of IL-1β and IL-6 in 24 hours samples and IL-10, IFN-α, IFN-γ and TNF-α in 48 hour samples.

### Statistical analyses

Subjects with CD4 cell counts below 100cells/μl were excluded from statistical analyses. All cytokine levels after stimulation were corrected for the amount of CD4 cells and the unstimulated levels. The production of cytokines was compared between groups using Kruskul-Wallis and Mann Whitney analyses. Results were found statistically significant at a level of 0.05, resulting in a p-value of 0.016 after Bonferroni adjustment for multiple tests. All statistical analysis was performed using the Statistical Product and Services Solutions package (SPSS) version 18.0 and GraphPad Prism version 5.0.

## Results

We included a total of 82 ART-naïve HIV-infected individuals with CD4 cell counts above 100cells/μl (**Table 1**). Overall, controls were slightly younger than the drug user groups (**Table 1**), but the groups did not differ in CD4 cell counts (p = 0.13, **Table 1**).

**Table 1:** Characteristics of ART-naïve HIV-infected individuals in this study (n = 82).

	**Heroin users(n = 17)**	**MMT(n = 17)**	**Former users(n = 17)**	**Controls(n = 31)**	**p-value** *
Male (%)	82.4	94.1	88.2	77.4	0.462
Median age (IQR)	32 (29–37)	33 (30–35)	32 (30–34)	28 (22–32)	0.006
Median Hb (IQR)	14.7 (13.2–16.0)	14.0 (12.9–15.1)	15.6 (14.8–16.2)	14.8 (13.2–15.9)	0.039
Median CD4 cell count (IQR)	411 (225–651)	240 (169–490)	377 (258–615)	283 (173–399)	0.13

Heroin users: used heroin in the last 30 days. MMT: individuals receiving methadone maintenance treatment (MMT) in the last 30 days. Former users: used heroin or methadone over one year ago. Controls: never used heroin or methadone.

*All groups were compared using Kruskal-Wallis analyses. ART: antiretroviral therapy, IQR: interquartile range; Hb: haemoglobin

Whole blood from these individuals was stimulated with various pathogens, namely *C*.*albicans*, *M*.*tuberculosis* and LPS isolated from *Escherichia coli*. The cytokine production of unstimulated samples was low and did not differ between groups; the median cytokine level was 32pg/μl for IL-1β (p = 0.466), 32.6pg/μl for IL-6 (p = 0.845), 6.7pg/μl for IFN-α (0.947), 4.0pg/μl for IFN-γ (0.431), 3.2pg/μl IL-10 (0.625) and 11.9 pg/μl for TNF-α (p = 0.382).

**Fig 1:**
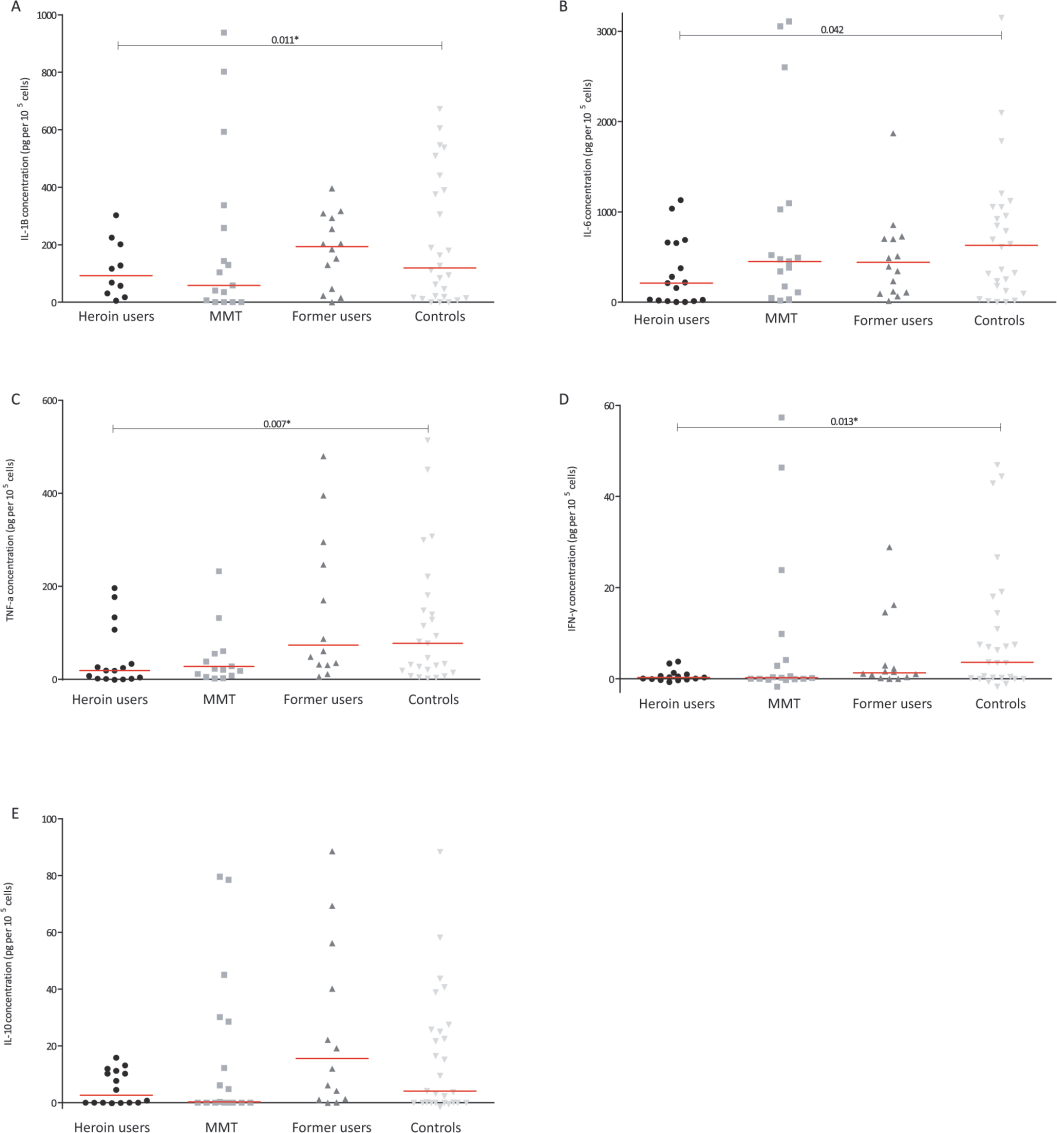
The concentration of IL-1B (A), IL-6 (B), TNF-α (C), IFN-γ (D) and IL-10 (E) after *ex vivo* stimulation with LPS of *E*.*coli* in various groups of ART-naïve HIV-infected individuals; individuals who used heroin in last 30 days (circles), individuals receiving methadone maintenance treatment (MMT: squares), individuals who used heroin over one year ago (former users: triangles) and those who never used opioids (controls: reversed triangles). The level of cytokines was corrected for baseline level and number of CD4 cells (and given in pictogram per 10^5^ cells). *Statistically significant difference (p< 0.016 after Bonferroni adjustment for multiple tests). MMT: methadone maintenance treatment; ART: antiretroviral therapy.

**Fig 2:**
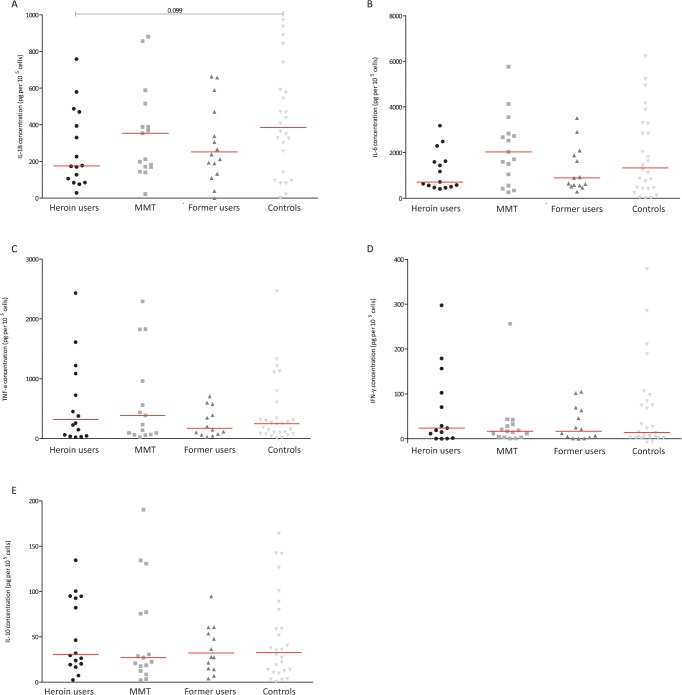
The concentration of IL-1B (A), IL-6 (B), TNF-α (C), IFN-γ (D) and IL-10 (E) after *ex vivo* stimulation with *Candida albicans* in various groups of ART-naïve HIV-infected individuals; individuals who used heroin in last 30 days (circles), individuals receiving methadone maintenance treatment (MMT: squares), individuals who used heroin over one year ago (former users: triangles) and those who never used opioids (controls: reversed triangles). The level of cytokines was corrected for baseline level and number of CD4 cells (and given in pictogram per 10^5^ cells). *Statistically significant difference (p< 0.016 after Bonferroni adjustment for multiple tests). MMT: methadone maintenance treatment; ART: antiretroviral therapy.

**Fig 3:**
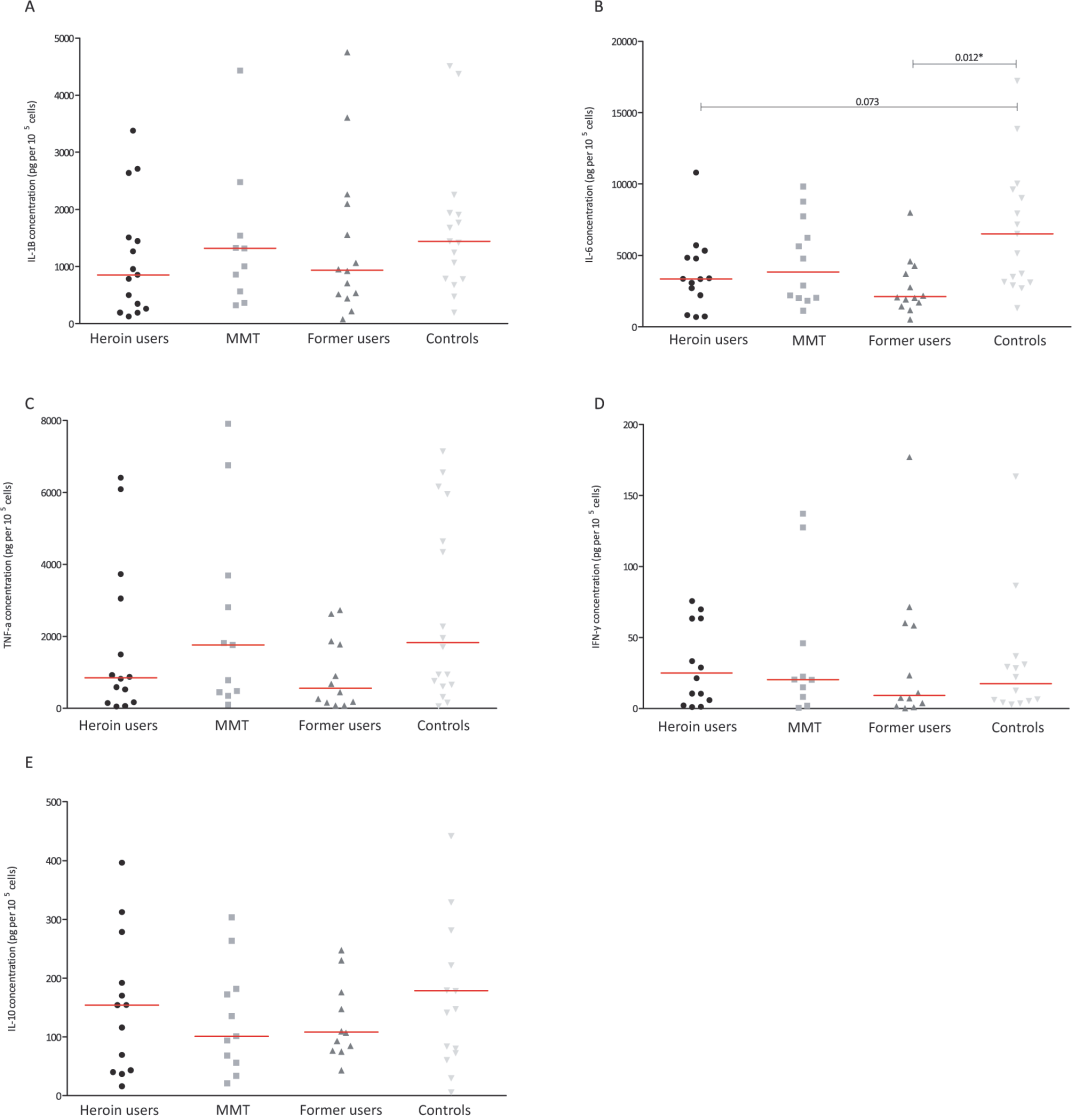
The concentration of IL-1B (A), IL-6 (B), TNF-α (C), IFN-γ (D) and IL-10 (E) after ex vivo stimulation with Mycobacterium tuberculosis in various groups of ART-naïve HIV-infected individuals; individuals who used heroin in last 30 days (circles), individuals receiving methadone maintenance treatment (MMT: squares), individuals who used heroin over one year ago (former users: triangles) and those who never used opioids (controls: reversed triangles). The level of cytokines was corrected for baseline level and number of CD4 cells (and given in pictogram per 10^5^ cells). *Statistically significant difference (p< 0.016 after Bonferroni adjustment for multiple tests). MMT: methadone maintenance treatment; ART: antiretroviral therapy.

The results from the stimulated samples showed that generally the cytokine production was significantly lower in heroin users (**Fig 1**) after exposure to LPS. Interesting the same was not observed after stimulation with *C*. *albicans* (**Fig 2**) and *M*. *tuberculosis* (**Fig 3**). Analysing the effect LPS exposure, significant less IL-1β, IL-6, TNF-α and IFN-γ production was found in heroin users (**Fig 1**). The production of these cytokines in MMT and previous users was comparable to the controls (**Fig 1**). No differences were found in the production of IL-10 after exposure to LPS. Stimulation with *C*. *albicans* from both heroin users and previous users seemed to produce less IL-1β and IL-6 than from controls; however these differences were not significant (**Fig 2**). Also the production of the other cytokines was comparable between all groups after exposure to *C*. *albicans*. Compared to controls, the IL-6 production after stimulation with *M*. *tuberculosis* was lower in heroin users (not significant p = 0.73) and previous users (p = 0.012) (**Fig 3**). Also the production of IL-1β and TNF-α seems to have the same trend, but the differences were not statistically significant. Overall, not clear differences were found after stimulation with *M*. *tuberculosis* (**Fig 3)**. The production of IFN-α was low in all stimulated samples and therefore these data are not shown.

## Discussion

In this study we show that the cytokine production is significantly down-regulated in HIV-infected heroin users compared to HIV-infected individuals who do not use opioids. Interesting, the altered cytokine production was only observed when cells were exposed to LPS but not to *M*. *tuberculosis* or *C*. *albicans*.

We found that LPS-induced cytokines were affected by heroin use, whereas *C*. *albicans* and *M*. *tuberculosis*-induced cytokines were not. One could argue that the receptors and pathways involved in candida (C-type lectin receptors) and tuberculosis (TLR 2 and NOD2) are less affected by opioids. However, previous studies have also found associations between opioids and these receptors [32–34]. In addition, the cytokine levels after stimulation with *M*. *tuberculosis* were relatively high and therefore might mask a difference between groups due to a ceiling effect. In the present study we only found effects of LPS, which is a specific agonist for TLR4; therefore our results suggest that opioids may affect mainly the TLR4-dependent cytokine production. A binding site for opioids on the TLR4 protein complex has indeed been demonstrated [35]. Studies have furthermore shown that both opioid agonists and antagonists alter the expression of TLR4 [36,37] or affect LPS signalling via the TLR4 signalling pathway [38], which strengthen the data of our study. The latter study showed that morphine alone only weakly increased TLR4 activation, but it significant inhibited TLR4 signalling after LPS stimulation. Interesting naltrexone (a μ-opioid antagonist) also significantly inhibited LPS-induced TLR4 activation [38]. Apart from its effect on monocyte/ macrophages, animal studies also indicate that morphine influences the production of TNF-α in mast cells in a TLR4-dependent manner [39]. Interestingly, the interaction between opioids and TLR4 may also work in the opposite direction, as activation of TLR4 can increase the production of endogenous opioids, such as β-endorphin [40]. Furthermore, some studies find that stimulation of TLR4 affects the opioid-induced responses, such as incubated cue-induced heroin-seeking and morphine tolerance [41,42], whereas others find no clear link [43]. Our study is in line with these studies, showing decreased signalling of TLR4 after LPS stimulation in individuals exposed to opioids. Even though we did not stimulate with the whole pathogen of *E*.*coli* we believe our results are still relevant to exposure to *E*.*coli*, as the TLR4 pathway is one of the most important pathways for this pathogen.

In addition to the mechanisms described above, bacterial translocation in opioid users might also contribute to our findings, as animal studies have shown that morphine increases bacterial translocation [44–46]. Opioid induced intestinal permeability may result in more exposure to microorganism and endotoxins; continuous exposure could result in the well-known process of endotoxin tolerance [47], which may explain our findings of reduced *ex vivo* production of pro-inflammatory cytokines after LPS exposure in heroin users. While endotoxin tolerance has been thought of as a protective mechanism against septic shock and ischaemia, its incidence is associated with high risks of secondary infections [48]. Various molecular mechanisms have been described that underlie the development of endotoxin tolerance, including defects in the TLR4 signalling pathway, activation of SOCS molecules, stimulation of anti-inflammatory cytokines such as IL-10, or epigenetic regulation [47–49]. In addition to opioids, HIV infection may increase gut permeability and exposure to endotoxins [50,51]. Our data show that response to LPS is diminished which could therefore be due to translocation of bacteria, especially since all study subjects were HIV positive.

In addition, the opioid-induced immunomodulation may be affected by other factors such as chronic hepatitis C infection. In this study we excluded individuals with signs of acute hepatitis but we did not test for (chronic) hepatitis C infection. Hepatitis C is highly prevalent among injecting drug users and we have previous shown that up to 90% of HIV-infected injecting drug users in Indonesia are seropositive for hepatitis C [52]. Previous studies have shown worse clinical outcomes among HIV-infected individuals with hepatitis C antibodies [53–58]. However, the relation between hepatitis C infection and HIV progression prior to ART remains unclear [53–56,59]; the effects of hepatitis C seems to be more pronounced among patients receiving ART [59,60]. A recent study examined the relation between hepatitis C and circulating cytokine levels [50]. Overall, cytokine levels were significantly higher in individuals co-infected with HIV and hepatitis C, while no data were presented about opioid use [50]. Since hepatitis C is strongly correlated with injecting drug use, it is therefore difficult to distinguish between the effects of opioids and hepatitis C.

Only one other study analysed the influence of heroin or morphine use on the cytokine response [30], showing that HIV-negative heroin users produce less IFN-γ and more IL-10 after stimulation with LPS. Interesting, this difference was larger for heroin users compared to morphine. In line with these results, we also found a decrease of IFN-γ after LPS stimulation; however we did not find an increase of IL-10. We found a lot of variance in the production of IL-10, which might have led to not finding a statistically significant difference. Furthermore, we also found a significant difference between heroin and methadone users. We have previous found similar results on the expression of HIV co-receptors [61]. Clinical and illicit opioids, such as morphine, methadone and heroin, exert their effects primarily through the μ-opioid receptor, but all with different affinity and some also bind to other opioid receptors [26–28,62]. In addition, multiple isotypes of the μ-opioid receptor exists, with differences in ligand binding affinity, rates of internalization and resensitisation. This may explain the differences that are observed when heroin, morphine or methadone are used.

Opioid-induced immunosuppression has shown to be mediated either directly via immune cells or via the central nervous system. In this study we focussed on the peripheral aspects of opioid-induced immunomodulation by using *ex vivo* stimulation of whole blood. However, the immune effects of opioids *in vivo* may also be affected by the central nervous system, as previously found in animal experiments [63,64]. The effects of opioids are especially important among HIV-infected individuals, as chronic opioid use has been shown to modulate HIV progression and facilitate HIV associated neurocognitive dysfunctions [52,65]. Among other aspects, opioids may modulate epigenetic and transcriptional regulation of the μ-opioids receptor; this may in turn alter the overall physiological effect of opioids which could be important in HIV disease progression [65].

In our study we determined drug use based on interviews and did not perform toxicological tests, which could perhaps result in a bias. However, we worked closely together with employees of a local NGO, who have been in close contact with our subjects for years. In addition, drug test will only show recent use of opioids (up to four days) and therefore might not be reliable in identifying active users that did not use in the previous couple of days. In addition, we do not have HIV viral loads of our study population, which might also affect the production of cytokines. However, we have previously shown that the viral load among people with and without a history of injecting drug use did not differ [52]. Another factor that may influence the cytokine production is HIV subtype, on which we have information in this population. In addition, drug users might have had a different immune status at inclusion. However, in unstimulated samples the cytokine levels were low in all groups. This indicates an inactive immune status of cells in all groups, and can therefore not explain the differences we show.

In conclusion, this is the first study to examine the immonumodulating effects of heroin and methadone in HIV-infected individuals. We show that heroin use decreases the cytokine response to LPS, but not to *M*. *tuberculosis* and *C*. *albicans*. Especially in HIV-infected individuals these immunomodulating effects of opioids may have deteriorating effects and increase the risk of secondary infections.
